# Serum interleukin-16 significantly correlates with the Vasculitis Damage Index in antineutrophil cytoplasmic antibody-associated vasculitis

**DOI:** 10.1186/s13075-020-02172-5

**Published:** 2020-04-07

**Authors:** Taejun Yoon, Jung Yoon Pyo, Sung Soo Ahn, Jason Jungsik Song, Yong-Beom Park, Sang-Won Lee

**Affiliations:** 1 Department of Medical Science, BK21 Plus Project, College of Medicine, Yonsei University, Seoul, Republic of Korea; 2 Division of Rheumatology, Department of Internal Medicine, College of Medicine, Yonsei University, 50-1 Yonsei-ro, Seodaemun–gu, Seoul, 03722 Republic of Korea; 3 Institute for Immunology and Immunological Diseases, College of Medicine, Yonsei University, Seoul, Republic of Korea

**Keywords:** Antineutrophil cytoplasmic antibody-associated vasculitis, Serum IL-16, Activity, Vasculitis damage, Reflect

## Abstract

**Background:**

Interleukin (IL)-16 is a T cell chemoattractant produced by peripheral mononuclear cells. We investigated whether IL-16 plays a pro- or an anti-inflammatory role in antineutrophil cytoplasmic antibody-associated vasculitis (AAV). Furthermore, we investigated whether the level of IL-16 could predict the activity and extent of organ damage in AAV based on AAV-specific indices.

**Methods:**

Seventy-eight patients with AAV from a prospective observational cohort were included in this analysis. Blood sampling and clinical assessments, including the Birmingham Vasculitis Activity Score (BVAS), Five-Factor Score (FFS), Short Form 36-item Health Survey (SF-36), and Vasculitis Damage Index (VDI), were performed, and laboratory data were collected. Serum IL-16 was measured from stored sera.

**Results:**

The median age was 62.0 years, and 27 patients were male. The median serum IL-16 concentration was 84.1 pg/dL, and the median BVAS, FFS, VDI, and SF-36 scores were 7.0, 1.0, 3.0, and 48.0, respectively. Among the AAV-related indices, the serum IL-16 concentration was correlated with VDI (*R*^2^ = 0.306, *P* = 0.006), but not with BVAS (*R*^2^ = 0.024, *P* = 0.834), FFS (*R*^2^ = − 0.069, *P* = 0.550), or SF-36 (*R*^2^ = − 0.015, *P* = 0.898). The serum IL-16 concentration also did not correlate with either the erythrocyte sedimentation rate or the C-reactive protein concentration. Per our analysis based on organ involvement, only patients with ear, nose, and throat manifestations had higher serum IL-16 concentrations relative to those with other conditions (*P* = 0.030).

**Conclusions:**

This was the first study to elucidate the clinical implication of serum IL-16 in patients with AAV. We found that the serum IL-16 level may reflect the cross-sectional VDI scores among AAV-specific indices. Future studies with larger numbers of patients and serial measurements could provide more reliable data on the clinical implications of serum IL-16 in AAV.

## Background

Antineutrophil cytoplasmic antibody (ANCA)-associated vasculitis (AAV) refers to a group of systemic vasculitides that affect small vessels, including microscopic polyangiitis (MPA), granulomatosis with polyangiitis (GPA), and eosinophilic granulomatosis with polyangiitis (EGPA) [1, 2]. In clinical practice, the following are often assessed at every visit: Birmingham Vasculitis Activity Score (BVAS), version 3 for activity; Five-Factor Score (FFS) for prognosis; the Korean version of the Short Form 36-item Health Survey (SF-36) for functional status; and the Vasculitis Damage Index (VDI) for end organ damage [3–6]. Although a single serum biomarker cannot replace each AAV-specific index, such a marker may have clinical implications as an immediate and convenient indicator if it reflects a specific AAV-related index. Therefore, the identification of a serologic biomarker that can estimate a cross-section of the many AAV-specific indices would be useful.

Interleukin (IL)-16 was first introduced as a T cell chemoattractant produced by peripheral mononuclear cells in 1982 [7]. This cytokine is produced by various cells, such as CD4+ T cells, CD8+ T cells, mast cells, eosinophils, and fibroblasts [8]. Bioactive IL-16 induces the chemotaxis of CD4+ TH1 cells by binding to CD4 to enhance T cell proliferation and stimulate B cell development, while inhibiting antigen-triggered TH2 cell-mediated inflammation and impairing macrophage inflammatory protein (MIP)-1beta and stromal cell-derived factor (SDF)-1alpha-mediated T cell chemoattraction [9]. Previous reports have highlighted the clinical significance of serum IL-16 in several typical systemic autoimmune diseases.

Serum IL-16 was observed to be significantly elevated in patients with systemic lupus erythematosus (SLE) relative to healthy controls, and the level of this cytokine was correlated with SLE activity [10]. Unlike SLE, the clinical role of serum IL-16 remains controversial in rheumatoid arthritis (RA). It was suggested that IL-16 production may be accelerated by IL-17 in RA and that serum IL-16 correlated with disease activity reflecting pro-inflammatory effect [11, 12]. However, there was a report that treatment with exogenous IL-16 inhibits the production of pro-inflammatory cytokines in rheumatoid synovitis, demonstrating the anti-inflammatory effects of IL-16 [13].

Because SLE, RA, and AAV share inflammatory mechanisms, we aimed to investigate whether serum IL-16 plays a pro- or an anti-inflammatory role in AAV. Furthermore, we wondered whether serum IL-16 might reflect the inflammatory burden of small pro-inflammatory molecules or reflect a consequence of extensive tissue damage. However, no previous study has elucidated the clinical implication of serum IL-16 in patients with AAV. In this study, we measured the serum IL-16 levels in patients with AAV and investigated the association of this factor with the cross-sectional scores of AAV-specific indices, such as the BVAS, FFS, SF-36, and VDI.

## Methods

### Patients

We included 78 patients with AAV in this study. The patients were enrolled in the Severance Hospital ANCA-associated VasculitidEs (SHAVE) cohort, a prospective observational cohort of patients with MPA, GPA, and EGPA, from November 2016 to May 2019. All patients were newly diagnosed with AAV at the Division of Rheumatology, Department of Internal Medicine, Yonsei University College of Medicine and Severance Hospital. The patients met the criteria of the 2007 European Medicine Agency algorithms for AAV and polyarteritis nodosa (the 2007 EMA algorithm) and the 2012 revised International Chapel Hill Consensus Conference Nomenclature of Vasculitides [1, 2]. At the time of diagnosis, patients with serious infections, including hepatitis B or C virus infection, malignancies, or secondary vasculitis features related to autoimmune diseases, were excluded from the SHAVE cohort. In addition, all patients were immunosuppressant-naïve at the enrollment of this study. This study was approved by the Institutional Review Board of Severance Hospital (4-2016-0901), and written informed consent was obtained from the patients at the time of blood sampling.

### Clinical and laboratory data

The collected demographic data included age and sex. The clinical manifestations were counted based on the items in BVAS version 3. We obtained the following laboratory data at diagnosis: perinuclear (P)-ANCA, cytoplasmic (C)-ANCA, myeloperoxidase (MPO)-ANCA, proteinase 3 (PR3)-ANCA, and acute-phase reactants, including erythrocyte sedimentation rate (ESR) and C-reactive protein (CRP). We also collected AAV-specific indices such as the BVAS version 3, FFS, SF-36, and VDI scores on the same day as the blood sampling and clinical assessment. According to the SHAVE cohort protocol, we obtained whole blood from each consenting patient, immediately isolated sera, and stored the samples at − 80 °C. Serum IL-16 concentrations in stored sera were measured using ELISA kits (Abcam, Cambridge, UK) according to the manufacturer’s instructions.

### Statistical analyses

All statistical analyses were conducted using SPSS software (version 25 for Windows; IBM Corp., Armonk, NY, USA). Continuous variables are expressed as means with standard deviations, and categorical variables are expressed as numbers (percentages). The correlation coefficient between the two variables was obtained using a Spearman correlation analysis. Significant differences in continuous variables between the two groups were compared using the Mann–Whitney test. *P* values < 0.05 were considered statistically significant.

## Results

### Characteristics of AAV patients

The baseline characteristics of the patients are described in Table 1. The median age was 62.0 years, and 27 patients were men. The median BVAS, FFS, and VDI scores were 7.0, 1.0, and 3.0, respectively.

**Table 1:** Characteristics of the clinical and laboratory data of 78 patients with AAV

Variables	Values
**Demographic data**	
Age (years)	62.0 (21.0)
Male sex (*N* (%))	27 (34.6)
**Variants (*N*(%))**	
MPA	40 (51.3)
GPA	21 (26.9)
EGPA	17 (21.8)
**AAV-specific indices**	
BVAS	7.0 (11.0)
FFS	1.0 (1.0)
VDI	3.0 (2.0)
SF-36 PCS score	48.0 (36.2)
SF-36 MCS score	57.0 (34.5)
**ANCA positivity (*N*(%))**	
MPO-ANCA positivity	43 (55.1)
PR3-ANCA positivity	7 (9.0)
ANCA negativity	28 (35.9)
**Clinical manifestations (*N*(%))**	
General	27 (34.6)
Cutaneous	9 (11.5)
Mucous membrane and eye	5 (6.4)
Ear, nose, and throat	36 (46.2)
Pulmonary	50 (64.1)
Cardiovascular	5 (6.4)
Abdominal	0 (0)
Renal	39 (50.0)
Nervous system	17 (21.8)
**Acute-phase reactants**	
ESR (mm/h)	37.5 (44.0)
CRP (mg/L)	2.6 (14.6)
**IL-16 (pg/mL)**	84.1 (198.7)

Values are expressed as medians (interquartile ranges, IQR) or numbers (percentages)

*AAV* ANCA-associated vasculitis, *ANCA* antineutrophil cytoplasmic antibody, *MPA* microscopic polyangiitis, *GPA* granulomatosis with polyangiitis, *EGPA* eosinophilic granulomatosis with polyangiitis, *BVAS* Birmingham Vasculitis Activity Score, *FFS* Five-Factor Score, *VDI* Vasculitis Damage Index, *SF-36* Short Form-36, *P* perinuclear, *C* cytoplasmic, *MPO* myeloperoxidase, *PR-3* proteinase 3, *AST* aspartate aminotransferase, *ALT* alanine aminotransferase, *ESR* erythrocyte sedimentation rate, *CRP* C-reactive protein

MPO-ANCA and PR3-ANCA were detected in 43 and 7 patients, respectively. The most common clinical manifestation was pulmonary (64.1%), followed by renal and ear, nose, and throat (ENT) manifestations. The median ESR and CRP levels were 37.5 mm/h and 2.6 mg/L, respectively. The median serum IL-16 level was 84.1 pg/mL.

### Correlation analysis

Of the AAV-specific indices, the serum IL-16 concentration correlated with the cross-sectional VDI scores (*R*^2^ = 0.306, *P* = 0.006), but not with the BVAS, FFS, or SF-36 scores or with ESR or CRP (Table 2).

**Table 2:** Correlation analysis of serum IL-16 with continuous variables in 78 patients with AAV

Variables	Correlation coefficient (***r***^2^)	*P* value
**AAV-specific indices**		
BVAS	0.024	0.834
FFS	− 0.069	0.550
VDI	0.306	0.006
SF-36 PCS score	− 0.015	0.898
SF-36 MCS score	− 0.122	0.288
**Acute phase reactants**		
ESR (mm/h)	− 0.034	0.769
CRP (mg/L)	− 0.007	0.952

*AAV* ANCA-associated vasculitis, *ANCA* antineutrophil cytoplasmic antibody, *BVAS* Birmingham Vasculitis Activity Score, *FFS* Five-Factor Score, *VDI* Vasculitis Damage Index, *SF-36* Short Form-36, *AST* aspartate aminotransferase, *ALT* alanine aminotransferase, *ESR* erythrocyte sedimentation rate, *CRP* C-reactive protein

### Comparative analysis

We compared the serum IL-16 concentration according to each organ or system wherein the involvement frequency exceeded 25%. Among the general, ENT, pulmonary, and renal manifestations, only patients with ENT manifestations exhibited a higher serum IL-16 concentration than those without ENT manifestations (*P* = 0.030; Table 3). In a correlation analysis of the serum IL-16 concentration and the total weighted score per each organ or system involvement, the former was correlated with only the total weighted score of ENT manifestations *(R*^*2*^ = 0.279, *P* = 0.013).

**Table 3:** Mean serum IL-16 levels based on organ or system involvement (frequency ≥ 25%) in AAV

Involved organ system	Absence	Presence	*P* value
General	167.4 ± 188.5	124.2 ± 137.6	0.297
Ear, nose, and throat	112.3 ± 142.5	199.2 ± 194.5	0.030
Pulmonary	147.0 ± 130.3	155.4 ± 194.0	0.839
Renal	151.0 ± 151.9	153.8 ± 193.7	0.945

Values are expressed as medians (interquartile ranges, IQR) or numbers (percentages)

*AAV* ANCA-associated vasculitis, *ANCA* antineutrophil cytoplasmic antibody, *MPA* microscopic polyangiitis, *GPA* granulomatosis with polyangiitis, *EGPA* eosinophilic granulomatosis with polyangiitis, *BVAS* Birmingham Vasculitis Activity Score, *FFS* Five-Factor Score, *VDI* Vasculitis Damage Index, *SF-36* Short Form-36, *P* perinuclear, *C* cytoplasmic, *MPO* myeloperoxidase, *PR-3* proteinase 3, *AST* aspartate aminotransferase, *ALT* alanine aminotransferase, *ESR* erythrocyte sedimentation rate, *CRP* C-reactive protein

Also, we compared the serum IL-16 concentration according to the ANCA status and specific disease type. IL-16 was significantly higher in patients with PR3-ANCA than those with MPO-ANCA or those without ANCA (*P* < 0.001; Table 4). IL-16 was higher in patients with GPA than those with MPA or EGPA although statistically not significant.

**Table 4:** Mean serum IL-16 levels based on disease type and ANCA status

Variables	Serum IL-16	*P* value
**Disease type**		
MPA	128.3 ± 151.2	0.162
GPA	213.9 ± 224.2	
EGPA	133.1 ± 136.0	
**ANCA status**		
MPO-ANCA positivity	133.1 ± 143.3	< 0.001
PR3-ANCA positivity	408.1 ± 275.0	
ANCA negativity	133.1 ± 143.3	

*ANCA* antineutrophil cytoplasmic antibody, *MPA* microscopic polyangiitis, *GPA* granulomatosis with polyangiitis, *EGPA* eosinophilic granulomatosis with polyangiitis, *MPO* myeloperoxidase, *PR3* proteinase 3

## Discussion

In this study, we demonstrated a correlation of the serum IL-16 concentration with cross-sectional VDI scores, but not with other AAV-specific indices. In addition, the serum IL-16 concentration differed significantly between patients with ENT manifestations and those without.

This study has raised many questions. For example, why does the serum IL-16 level correlate with only the cross-sectional VDI scores? The first hypothesis is that serum IL-16 might be associated with fibrotic changes. Compared to BVAS, FFS, or acute-phase reactants, the VDI of AAV may be more strongly associated with chronic inflammation (late phase or fibrotic phase) than with acute inflammation (early phase or pro-fibrotic phase) [14]. Kazuo et al. reported that IL-16 levels correlated with skin involvement in patients with systemic sclerosis reflecting the role of IL-16 in skin fibrosis [15]. Furthermore, there was a study showing that pro-fibrotic mediators induce IL-16 upregulation and IL-16 gene expression was elevated in lung tissue from patient with idiopathic pulmonary fibrosis [16]. Therefore, we assume that the positive correlation between serum IL-16 and VDI may reflect the extent of end organ damage due to progressive fibrosis.

The second hypothesis is that serum IL-16 might be associated with the secondary necrosis of neutrophils. Considering the importance of neutrophil in pathogenesis of AAV, we focused on the relation between IL-16 and neutrophil. It was previously revealed that in neutrophils, IL-16 was stored in a preformed form and that it was passively released during secondary necrosis rather than being actively released from viable or apoptotic cells [17]. Under normal circumstances, neutrophils have a short lifespan and are cleared via apoptosis once their function has ended. However, apoptotic neutrophils may not be properly cleared in chronic inflammatory situations, and secondary necrosis may occur. We assumed that in late-phase AAV, accumulated neutrophils undergo secondary necrosis and secrete IL-16, which causes tissue damage. This process may explain the positive correlation between serum IL-16 and VDI of AAV.

The association between ENT involvement and serum IL-16 may be secondary to the phase difference between acute inflammation (early phase or pro-fibrotic phase) and chronic inflammation (late phase or fibrotic phase). We therefore analyzed the sub-items of four frequent manifestations per phase. Regarding the general manifestations, all sub-items such as myalgia, arthralgia, fever, and weight loss may be closely related to the acute phase. Regarding the pulmonary manifestations, five of seven sub-items may be somewhat related to the acute phase. Likewise, regarding the renal manifestations, four of five sub-items may also be related to acute inflammation or exacerbation. Conversely, with respect to ENT manifestations, four of five sub-items, such as paranasal sinusitis (thickened sinus wall), subglottic stenosis, conductive hearing loss, and sensorineural hearing loss, tend to be related to the relatively chronic phase of AAV [3].

However, there is a possibility that the degree of inflammatory burden of renal and lung involvement in our patient population was not very high. Despite 50.0% and 64.1% of our patients had renal and pulmonary involvement, our patient’s median BVAS was 7.0, implying that the inflammation of renal and pulmonary manifestation was not highly active. This could partly be the factor that serum IL-16 correlating only with ENT involvement. Further research with larger population is needed to clarify the relationship between serum-IL-16 and organ involvement.

Another hypothesis regarding the association of serum-IL16 with ENT involvement is that IL-16 might have an association with PR3-ANCA. PR3-ANCA tends to have dominant ENT involvement and is associated more with the GPA than MPA or EGPA. Serum IL-16 in our study was significantly higher in patients with PR3-ANCA. One possible explanation for this phenomenon is that IL-16 could play a role in causing granuloma. There was a report showing that high levels of IL-16 were seen in immunohistochemical staining of sarcoidosis granuloma [18]. Regarding that the PR3-ANCA is more relevant in GPA than in MPA, there is a possibility of IL-16 being associated with granuloma. However, further research is needed to understand the relationship between serum IL-16 and PR3-ANCA.

One strength of this study is that it is the first to demonstrate an association of serum IL-16 with cross-sectional VDI scores, but not with BVAS or FFS scores or acute -phase reactants. Furthermore, we excluded patients with other underlying serious medical conditions, and IL-16 was measured from the stored sera which was drawn before starting immunosuppressant drugs, which can minimize the confounding factors. However, our study also has several limitations. First, the sample size was not large enough to ensure that the findings are generalizable to all patients with AAV. Second, we cannot provide serial or paired results, despite the inclusion of patients with AAV in the prospective cohort. Third, we could not assess the duration from symptom to diagnosis, which might be informative to evaluate the acute or chronic status due to the complexity of the disease.

## Conclusions

In conclusion, the serum IL-16 level may reflect the cross-sectional VDI scores among AAV-specific indices. Future studies with larger numbers of patients and serial measurements could provide more reliable data on the clinical implications of serum IL-16 in AAV.

## Data Availability

The datasets used and/or analyzed during the current study are available from the corresponding author on reasonable request.

## Abbreviations

AAV: Antibody-associated vasculitis
ANCA: Antineutrophil cytoplasmic antibody
BVAS: Birmingham Vasculitis Activity Score
CRP: C-reactive protein
EGPA: Eosinophilic granulomatosis with polyangiitis
ENT: Ear, nose, and throat
ESR: Erythrocyte sedimentation rate
FFS: Five-Factor Score
FVSG: French Vasculitis Study Group
GPA: Granulomatosis with polyangiitis
MPA: Microscopic polyangiitis
RA: Rheumatoid arthritis
SHAVE: Severance Hospital ANCA-associated VasculitidEs
SLE: Systemic lupus erythematosus
VDI: Vasculitis Damage Index
